# Epigenetic remodeling in preimplantation embryos: cows are not big mice

**DOI:** 10.21451/1984-3143-AR2018-0068

**Published:** 2018-09-06

**Authors:** Pablo J. Ross, Rafael V. Sampaio

**Affiliations:** 1 Department of Animal Science, University of California Davis, Davis, CA, United States.; 2 Department of Veterinary Medicine, Faculty of Animal Science and Food Engineering, University of Sao Paulo, Pirassununga, Sao Paulo, Brazil.

**Keywords:** bovine, epigenetics, embryo, preimplantation development, histone modifications, DNA methylation

## Abstract

Epigenetic mechanisms allow the establishment and maintenance of multiple cellular phenotypes from a single genomic code. At the initiation of development, the oocyte and spermatozoa provide their fully differentiated chromatin that soon after fertilization undergo extensive remodeling, resulting in a totipotent state that can then drive cellular differentiation towards all cell types. These remodeling involves different epigenetic modifications, including DNA methylation, post-translational modifications of histones, non-coding RNAs, and large-scale chromatin conformation changes. Moreover, epigenetic remodeling is responsible for reprogramming somatic cells to totipotency upon somatic cell nuclear transfer/cloning, which is often incomplete and inefficient. Given that environmental factors, such as assisted reproductive techniques (ARTs), can affect epigenetic remodeling, there is interest in understanding the mechanisms driving these changes. We describe and discuss our current understanding of mechanisms responsible for the epigenetic remodeling that ensues during preimplantation development of mammals, presenting findings from studies of mouse embryos and when available comparing them to what is known for human and cattle embryos.

## Introduction

The simplicity of the morphological changes that occur during early embryo development, mostly cleavage division at initial stages, masks the molecular events that underlie the profound and dynamic remodeling of the embryonic transcriptome and epigenome during this period. Pre-implantation development in all animal species encompasses unique features, such as drastic transcriptional and epigenetic remodeling ( Bogliotti and Ross, 2015 ). Epigenetic information, in the form of histone modifications and DNA methylation, is generally stable, due to its capacity to be inherited from cell to cell after mitosis; and flexible, since it can be modified, e.g., during cellular differentiation. The epigenetic information of the sperm and oocyte is extensively remodeled with formation of the embryo and this remodeling is likely critical to generate the proper pattern of embryo gene expression required for continued development. Interestingly, some genomic features escape epigenetic erasure in the embryo, e.g., DNA methylation of imprinting marks and some retrotransposons ( Messerschmidt, 2012 ).

The early stages of pre-implantation development occur in the absence of transcription and development relies on maternal proteins and mRNAs stored in the cytoplasm of the oocyte during oocyte growth and maturation ( Tadros and Lipshitz, 2009 ). The transition from maternal to embryonic control of development includes the degradation of maternal products and the activation of the embryonic genome (EGA). EGA is marked by a massive transcription from the embryonic genome that is vital for further embryonic development. EGA occurs in a species-specific timing: in mice at the early 2-cell stage ( Schultz, 1993 ), in pigs at the 4-cell stage ( Jarrell *et al*., 1991 ), and in humans and cattle at the 8-cell stage ( Braude *et al*., 1988 ; Memili and First, 2000 ; Graf *et al*., 2014 ). Evidence suggests that the drastic epigenetic remodeling observed during early development is needed for the correct activation of the embryonic genome. Nonetheless, the mechanisms and the identity of genes remodeled during this critical developmental period in most mammalian species are largely unknown.

## Epigenetic remodeling during early development

The epigenetic information of sperm and oocytes is extensively remodeled with formation of totipotent blastomeres ( Zhou and Dean, 2015 ). This remodeling is thought necessary to reset the epigenetic status of the differentiated gametic genomes into a totipotent embryonic state to support a pattern of gene expression required for successful development. While this extensive epigenetic remodeling takes place, some genomic features escape epigenetic erasure in the embryo, e.g., imprints and some retrotransposons ( Messerschmidt, 2012 ). A large part of this reprogramming is driven by oocyte factors of maternal origin. The capacity of the oocyte to “induce” epigenetic reprograming is best evidenced in the case of somatic cell nuclear transfer (SCNT), where a somatic cell nucleus is stripped-off its epigenetic-enforced cell fate and made amenable to drive the full developmental program. While sometimes complete, epigenetic reprogramming after SCNT is not always fully achieved resulting in inefficiencies associated with cloning animals by nuclear transplantation. Thus, SCNT/cloning represents an excellent model to understand epigenetic mechanisms, differentiation, and reprogramming ( Long *et al*., 2014 ).

At the molecular level, epigenetic information is represented mainly by DNA methylation and posttranslational histone modifications. While global changes in epigenetic information during preimplantation development and after SCNT have been studied, much less is known about the locus-specific changes of these epigenetic marks across the genome. The current available data is mostly for mice, and while informative, some differences in development between mice and livestock species indicate that it will be important to gather species-specific knowledge if a clear understanding of early development is desired, which could fuel applications such as *in vitro* embryo production, SCNT, and epigenetic selection and editing for improved phenotypes.

Although there are more research resources and tools for mouse than for other mammalian species, substantial advances in current genomic technologies have effectively leveled the playing field for many other species, such as cattle. The advent of sequencing technologies to determine transcriptomic and epigenomic features have demonstrated that similar information can be readily collected in any species for which a high quality and well annotated genome exists, e.g., cattle, sheep, pigs. Furthermore, siRNA and gene editing technologies like CRISPR/Cas9 now allow generation of knockdown (KD) and knockout (KO) embryos/animals, respectively, in almost any species. For modeling human development, cattle preimplantation embryos have similarities to human in areas in which mice differ, such as a similar timing for genome activation and reprogramming gene expression, and a more similar genome sequence and organization ( Bovine Genome Sequencing and Analysis Consortium, 2009 ). For example, a recent comparison of RNA-seq data between human, mouse, and cattle embryos across different stages of preimplantation development found more similarities in the transcriptomes between bovine and human than mouse and human, indicating that bovine embryos are an excellent model to study human preimplantation development ( Jiang *et al*., 2014 ).

It is quite possible that species differences in timing of the major EGA, when the dramatic reprogramming in gene expression occurs and is essential for further development, could reflect differences in epigenetic remodeling leading to EGA. In mice, EGA occurs during first cell cycle ( Schultz, 1993 ; Hamatani *et al*., 2004 ) and is characterized by a widespread promiscuous production of unprocessed transcripts that precedes the major period of EGA ( Abe *et al*., 2015 ), which is associated with an open chromatin state ( Wu *et al*., 2016 ). In contrast, the major EGA occurs after 3-4 cell cycles (8/16 cell stage) in cattle and human embryos, which can develop to the 8/16-cell stage in the absence of embryonic transcription ( Camous *et al*., 1986 ; Kopecny, 1989 ), although transcription is detectable in 2- and 4-cell stage bovine embryos ( Viuff *et al*., 1996 ; Memili *et al*., 1998 ).

Also, differences between mouse and human embryos are apparent during the first embryo differentiation events. Single-cell analysis in human embryos revealed marked differences between human and mouse embryos with respect to lineage specification in the early embryos and X-chromosome inactivation (XCI) ( Petropoulos *et al*., 2016 ). Whereas mouse embryonic cells segregate first into inner cell mass (ICM) and trophectoderm (TE) and then the ICM cells differentiate into epiblast (EPI) and primitive endoderm (PE), in human embryos the first differentiation event leads to the simultaneous formation of EPI, PE, and TE lineages, with some earlier cells co-expressing markers for all three lineages ( Petropoulos *et al*., 2016 ). In terms of XCI, whereas mouse embryos undergo imprinted inactivation of the paternal X-chromosome prior to the blastocyst stage, human embryos express both chromosomes and accomplish dosage compensation by down-regulating gene expression levels ( Petropoulos *et al*., 2016 ). Recent application of CRISPR/Cas9 technology to human and cattle embryos has highlighted different consequences of OCT4 gene inactivation for these species compared to mice. Generation of KO embryos by direct injection of CRISPR/Cas9 in human ( Fogarty *et al*., 2017 ) or cattle embryos ( Daigneault *et al*., 2018 ) or by SCNT from CRISPR/Cas9 edited bovine fibroblasts ( Simmet *et al*., 2018 ), showed similarities in the role of OCT4 in bovine and human embryos, while differing from results obtained in OCT4-KO mice.

## Chromatin conformation changes during preimplantation development

Alterations in chromatin structure due to for example histone modifications, modulate transcription by allowing or restricting transcription factors access to genome regulatory elements. Generally, chromatin organization and TF binding dictate the impact of regulatory elements on gene expression ( Kouzarides, 2007 ; Schep *et al*., 2015 ). Since regulatory regions, like promoters and enhancers, are generally more accessible ( Gross and Garrard, 1988 ), mapping open chromatin can identify potential regulators based on sequence motif analyses ( Buenrostro *et al*., 2013 ; Lara-Astiaso *et al*., 2014 ; Lavin *et al*., 2014 ). Assays to map open chromatin at a genome-wide level have been developed and recently optimized for low cell numbers. DNase-seq and ATAC-seq can be performed with as little as a 100 cells ( Buenrostro *et al*., 2013 ; Buenrostro *et al*., 2015 ; Cusanovich *et al*., 2015 ) and have been applied to mouse early embryos ( Lu *et al*., 2016 ; Wu *et al*., 2016 ). ATAC-seq data has shown that open chromatin regions develop as clusters and are enriched for retrotransposon genes. Importantly, these open chromatin regions disappear in the presence of the transcriptional inhibitor α-amanitin, indicating that chromatin opening is transcription dependent ( Wu *et al*., 2016 ). DNase-seq of mouse preimplantation embryos has shown that expressed genes are associated with open chromatin regions, and that inactive genes associated with open chromatin are activated at later developmental stages ( Lu *et al*., 2016 ), indicating a poised chromatin status. Also, detection of chromatin organization by Hi-C methodology indicated that the often-conserved higher order chromatin associations are disorganized in mouse MII oocytes and become established as embryos initiate gene expression ( Flyamer *et al*., 2017 ). Application of global chromatin accessibility assays to bovine preimplantation embryos could provide important information towards understanding the dynamics of nuclear reprogramming in species with delayed embryonic genome activation.

## DNA methylation remodeling during preimplantation development

DNA methylation is an epigenetic modification essential for normal mammalian development ( Li *et al*., 1992 ; Okano *et al*., 1999 ). DNA methylation consists of the addition of a methyl group to the fifth carbon position of cytosine residues in the DNA (5-mC), catalyzed by DNA methyltransferases (DNMT1 for maintenance and DNMT3A and DNMT3B for de novo methylation). DNA methylation exert its effects by blocking access to genome regulatory regions, but also by recruiting transcriptional repressors and/or chromatin modifiers to a specific genome location. In general, DNA methylation is associated with transcriptional repression ( Schultz *et al*., 2015 ); however, this simplistic view is not always the case, and DNA methylation can be associated with different gene expression states depending on the genomic context. For example, it has been observed that gene body DNA methylation is often indicative of active transcription ( Hellman and Chess, 2007 ; Cotton *et al*., 2009 ; Kobayashi *et al*., 2012 ; Schroeder *et al*., 2015 ), as is the case in oocytes and placental tissue of different mammals including cattle ( Schroeder *et al*., 2015 ).

It has been well established that the levels of DNA methylation, which are relatively high in sperm and at intermediate levels in the oocyte, decrease during preimplantation development. Early immunostaining data indicated that methylated cytosines were rapidly and actively removed from the paternal genome, while a gradual, replication dependent passive removal occurred at the maternal genome ( Mayer *et al*., 2000 ). The discovery of TET mediated 5-mC oxidation to 5-hydroxymethyl cytosine (5-hmC) helped clarify the reasons for rapid 5-mC immunoreactivity disappearance from the male PN, as a result of a remarkable global conversion of 5-mC to 5-hmC primarily at the paternal genome. Thus, active DNA demethylation of the paternal genome has been ascribed to TET activity ( Iqbal *et al*., 2011 ). However, it is important to highlight that 5-hmC is a biochemically methylated cytosine, although at an oxidized state, and that further processing would be required for getting an unmethylated cytosine in the same position ( Gkountela and Clark, 2014 ). On the other hand, since 5-hmC is only poorly recognized by DNMT1, its presence can lead to passive demethylation, by preventing maintenance methylation. Indeed, 5-hmC labeling of chromosome spreads in blastomeres from zygote to 8-cell embryo showed that this mark is mainly localized at the paternal chromosomes, and most of those genomic regions were demethylated in a DNA replication dependent manner ( Inoue and Zhang, 2011 ).

The differential activity of TET3 on the male and female derived genomes results from the protective effect of DPPA3 (a.k.a. STELLA/PGC7). DPPA3 is one of the most abundant transcripts in oocytes and protects 5-mC from TET3-mediated conversion to 5-hmC by binding to chromatin containing H3K9me2, which is abundant in the oocyte and but mostly lacking in sperm, with some H3K9me2 observed in paternally imprinted genes of the mature sperm ( Nakamura *et al*., 2012 ). Although DPPA3 has a very low amino acid sequence conservation between mouse, human and cows (~30% identity) Payer *et al*., 2003; Thelie *et al*., 2007), the function of protecting the female genome from TET3 activity is conserved across these species ( Bakhtari and Ross, 2014a ).

In mice, the mechanisms for protecting imprinted genes from replication-induced passive demethylation have been well characterized and ZFP57. This protein recognizes a methylated hexanucleotide sequence present at imprinted control regions and associates with TRIM28 (a.k.a. KAP1), resulting in recruitment of DNMT1 to the imprinted control region and therefore maintaining the methylation status at the imprinted control region after DNA replication ( Messerschmidt *et al*., 2012 ). Interestingly, ZFP57 is not expressed in human or cow oocytes, suggesting that other mechanisms for protection of imprinting must exist in these species ( Okae *et al*., 2014 ). The oocyte-specific DNMT1o is mainly located at the cytoplasm of preimplantation embryonic blastomeres and enters the nucleus only at the 8-cell embryo stage ( Howell *et al*., 2001 ).

Recent studies suggest a big role for DNA replication dependent (passive) demethylation, either from a native (5mC) or oxidized (5hmC) form; however, a small contribution for active demethylation cannot be excluded. Such active demethylation, if present would only be minor. Importantly, the role of Thymine DNA Glycosylase (TDG) in active demethylation in the zygote was discarded by studying mutant mice, implying that other enzymatic activity could be responsible ( Gkountela and Clark, 2014 ).

DNA demethylation is necessary for epigenetic reprogramming of the somatic nuclei ( Simonsson and Gurdon, 2004 ); and is partly mediated by TET activity ( Gu *et al*., 2011 ). However, donor cell DNA is often only partially demethylated ( Reik *et al*., 2001 ), resulting in cloned embryos with increased DNA methylation levels when compared to fertilized ones ( Wossidlo *et al*., 2011 ).

Imprinted genes are regulated by parental specific DNA methylation and are often altered during cloning ( Smith *et al*., 2012 ) and other assisted reproductive technologies (ART) in cattle ( Smith *et al*., 2015 ), as well as in humans ( Nelissen *et al*., 2014 ). Alterations of the epigenetic control of imprinted genes during the *in vitro* embryo development, have been suggested as the main reason for the appearance of the Large Offspring Syndrome (LOS) ( Young *et al*., 1998 ; Young *et al*., 2001 ). In humans, imprinted genes alterations during ARTs have been associated to the increased occurrence of syndromes including Beckwith-Wiedemann, Prader-Willi, Russell-Silver, and Angelman ( Amor and Halliday, 2008 ).

**Figure 1:**
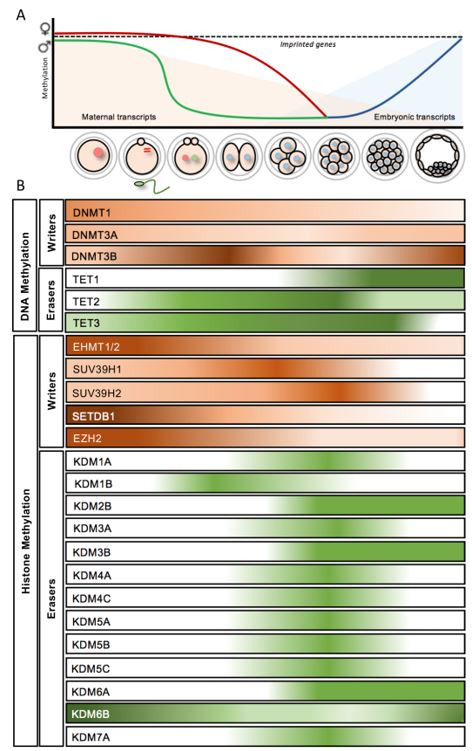
Epigenetic landscape in bovine preimplantation embryos. (A) Dynamics of DNA methylation levels and embryonic genome activation. (B) Global levels of epigenetic *writers* and *erasers* during bovine preimplantation embryo development.

In bovine, the presence of DNMT3A, DNMT3B, and DNMT3L during oocyte growth is related to the establishment of imprinted genes ( O'Doherty *et al*., 2012 ). During subsequent phases of development, whereas DNMT1 and DNMT3A are present ( Golding *et al*., 2011 ), it seems that DNMT3B is the major responsible for the control of methylation levels ( Dobbs *et al*., 2013 ). Besides these methylation writers, the dynamic of the main erasers has been also described in bovine development. The expression of TET family is also required for demethylation process ( Bakhtari and Ross, 2014b ; Figure 1 ).

## Histone modification remodeling during preimplantation development

In Eukaryotes, the DNA is packaged in chromatin inside the nucleus. The nucleosome constitutes the basic unit of chromatin and consists of a segment of DNA (~147bp), wrapped twice around an octamer of histone core proteins (two copies of: H2A, H2B, H3, and H4) ( Kornberg, 1974 ). The amino terminal (N-terminal) portions of the histone proteins remain outside of the nucleosome core and can be subject to post-translational modifications ( Luger and Richmond, 1998 ). Histone modifications can include phosphorylation, ubiquitylation, sumoylation, acetylation, and methylation, among others. Histone modifications can have different consequences for chromatin compaction and accessibility as well as being recognized by different transcription factors and regulators; thus, histone modifications can have varied effects on gene expression. In general, histone acetylation is associated with a more relaxed chromatin state that is permissive for gene expression. Histone methylation can take place at arginine (R) or lysine (K) residues. Methylation at lysine residues is one of the most studied marks and can signal either activation or repression, depending on the sites of methylation and the number of methyl groups (mono- (me1), di- (me2) or tri- (me3)), which are added in a stepwise progressive manner. Histone methylation is generally regarded as a relatively stable epigenetic mark, with the rate of histone methyl group turnover similar to that of histone turnover ( Bannister *et al*., 2002 ; Margueron *et al*., 2009 ).

Sperm chromatin is unique in that most histone proteins are replaced by protamines ( Braun, 2001 ). Upon fertilization, protamines are rapidly exchanged with maternal histones that subsequently become methylated at position H3K4. On the other hand, oocyte DNA is wrapped around modified histones, e.g., H3K9me2/3, H4K20me3, H3K36me3, H3K27me3 and H3K64me3. These differences create an asymmetry in epigenetic signatures of maternal and paternal genomes readily observed by immunostaining of PN-stage embryos, and persist in 2-cell embryos ( Lepikhov *et al*., 2008 ). How this asymmetry impacts gene expression is not known. Furthermore, in humans, an estimated 5-15% of the sperm DNA is associated with histones bearing specific modifications ( Gatewood *et al*., 1987 ; Hammoud *et al*., 2009 ), and some sperm histones may contribute to gene regulation during early development ( van der Heijden *et al*., 2009 ). In mice, over-expressing a histone demethylase during spermatogenesis results in increased levels of H3K4me2 and RNA in the sperm and impaired offspring health for the next 3 generations, suggesting that alterations to the sperm epigenome has transgenerational effects ( Siklenka *et al*., 2015 ). However, it is not clear to what extent paternal histones are inherited by the offspring and contribute to embryonic chromatin.

During epigenetic remodeling of bovine embryos, few histone methyltransferases are in charge to ensure the correct maintenance of the epigenome. The most characterized *writers* are EHMT1/2, SUV39H1/H2, SETDB1 and EZH2, which are responsible for the methylation of H3K9me2, H3K9me3, and H3K927me3, respectively ( McGraw *et al*., 2007 ; Ross *et al*., 2008 ; Golding *et al*., 2015 ; Zhang *et al*., 2016; Fig. 1 ).

Global levels of the repressive H3K27me3, H3K64me3, and H4K20me3 marks, highly abundant on the maternal genome, decrease after fertilization but re-establish to oocyte levels by the blastocyst stage ( Ross *et al*., 2008 ; Daujat *et al*., 2009 ; Wongtawan *et al*., 2011 ). Loss of these repressive marks is driven by active mechanisms, as opposed to passive dilution with each cell division, because inhibiting DNA replication with aphidicolin does not prevent the decrease in H3K64me3 ( Daujat *et al*., 2009 ) or H3K27me3 ( Canovas *et al*., 2012 ). Expression of enzymes responsible for removal of the methylation marks from H3K4 (KDM1A, KDM1B, KDM2B, KDM5A, KDM5B and KDM5C), H3K9 (KDM3A, KDM3B, KDM3C, KDM4A, KDM4B and KDM4C), and H3K27 (KDM6A, KDM6B and KDM7A) were recently characterized in bovine early development ( Glanzner *et al*., 2018 ; Fig. 1 ).

In cattle, H3K27me3 is removed during cleavage divisions catalyzed by KDM6B (JMJD3) activity. Down-regulation of KDM6B in cattle oocytes, which prevents the decrease in H3K27me3, results in impaired EGA and reduced development to blastocyst, in both parthenogenetic ( Canovas *et al*., 2012 ) and fertilized ( Chung *et al*., 2017 ) embryos.

In mouse, KDM6B depletion in preimplantation embryos alters H3K27me3, preventing CDX2 and GATA3 expression from the embryonic genome and results in improper TE development and implantation failure ( Saha *et al*., 2013 ). Similarly, deletion of JMJD2C, a demethylase specific for the repressive H3K9me3 mark, causes arrest of development before the blastocyst stage ( Wang *et al*., 2010 ). Furthermore, down-regulation of KDM1A, a demethylase with activity towards H3K4me1/2 and H3K9me2, results in increased H3K9me3 and H3K4me1/2/3 levels and impaired genome activation with developmental arrest at the 2-cell stage in mouse ( Ancelin *et al*., 2016 ). These studies highlight the important role for the active removal of repressive histone marks in reactivating gene expression and further embryo development.

Acquisition of activating epigenetic marks, such as H3K4me3, is also critical for development. Deletion of Mll2, which encodes an H3K4 methylases results in 2-cell stage arrest in mouse ( Andreu-Vieyra *et al*., 2010 ). Similarly, overexpression of a K-to-M mutant histone H3, which cannot be methylated at K4, results in a decreased level of minor activation of the paternal genome and subsequent major EGA, decreasing preimplantation development ( Aoshima *et al*., 2015 ). Furthermore, gene inactivation is also regulated by the absence or removal of activating marks. For example, using ChIP and qPCR, loss of H3K4m3 rather than acquisition of H3K9me3 was associated with retrotransposon silencing in mouse embryos ( Fadloun *et al*., 2013 ). Absence of H3K4me3 demethylase (KMD1A) in oocytes leads to deficient suppression of LINE-1 retrotransposon expression. Similarly, knock down of KDM5B (specific for H3K4me2/3) in pig ( Huang *et al*., 2015 ) and mouse ( Dahl *et al*., 2016 ; Zhang *et al*., 2016a ) embryos results in increased H3K4me3 and decreased preimplantation development.

H3K9 methylation has been implicated as an important barrier affecting SCNT reprogramming efficiency ( Chen *et al*., 2013 ; Matoba *et al*., 2014 ; Ng and Gurdon, 2014 ). In cattle embryos, methylation of H3K9 is remodeled in parallel with DNA methylation in normal embryos and often displays hypermethylation in cloned embryos, mirroring the case of DNA methylation ( Santos *et al*., 2003 ). It has been suggested that both DNA methylation and H3K9 methylation are largely refractory to the oocyte reprogramming potential ( Santos *et al*., 2003 ).

A combination of transcriptome analysis of mouse SCNT and fertilized embryos at MET and histone ChIP-seq in the donor cells allowed the identification of “reprogramming resistant regions” (RRR) ( Matoba *et al*., 2014 ). These RRR were enriched for H3K9 methylation, supporting the evidence that H3K9 methylation are a major hindrance to nuclear reprogramming. Strikingly, silencing of histone methyltransferase enzymes by siRNA in the donor cells or by transiently overexpressing H3K9 demethylases by mRNA injection in cloned embryos was able to reactivate reprogramming resistant regions genes and dramatically increase mouse SCNT efficiency ( Matoba *et al*., 2014 ). Importantly, the application of the approach to reduce H3K9me3 during SCNT was used for producing the first monkey from SCNT ( Liu *et al*., 2018b ).

Multiple strategies have been suggested to surpass the reprogramming barrier formed by H3K9me3; the most widely attempted approach being the treatment of donor somatic cells with histone deacetylases or methyltransferases inhibitors ( Kishigami *et al*., 2006 ; Martinez-Diaz *et al*., 2010 ; Akagi *et al*., 2011 ). However, results are controversial, showing promising results for species such as mice ( Kishigami *et al*., 2006 ) and porcine ( Zhao *et al*., 2009 ), while similar approaches in bovine embryos have yielded inconsistent results ( Sangalli *et al*., 2012 ; Sangalli *et al*., 2014 ). In cattle, recent publications focusing on H3K9 methylation reported promising results on nuclear reprogramming, showing that two different approaches could be used to improve blastocyst rates, including inhibiting H3K9 methyltransferases or injecting H3K9 demethylases in NT embryos ( Zhang *et al*., 2017 ; Liu *et al*., 2018a ).

Recent development of low-input ChIP-seq methodologies has allowed capturing the locus-specific whole genome localization of some histone modifications during early mouse development ( Dahl *et al*., 2016 ; Liu *et al*., 2016 ; Zhang *et al*., 2016a ). These studies observed unusually broad genomic domains of H3K4me3 in oocytes and early embryos, which transitioned to the more common tight localization at the transcription start sites of active genes in later stage embryos ( Dahl *et al*., 2016 ; Liu *et al*., 2016 ; Zhang *et al*., 2016a ). The relationship between the unusual H3K4me3 pattern and activation of gene expression is not yet understood.

Large amounts of critical information can be obtained from studying the epigenome of early embryos. In the animal production field, such information could be useful, for example, for interpreting aberrant epigenetic landscapes observed when using some assisted reproductive technologies, such as SCNT. For the biology field, the information is significant for understanding how genes are regulated in a pluripotent state, during de-differentiation (from gametes to pluripotent blastomeres), and during re-differentiation (early lineage commitment).

Recently, the derivation of bovine embryonic stem cells ( Bogliotti *et al*., 2018 ) opened an opportunity at comparing the histone methylation profiles in bovine pluripotent stem cells to that of human and mouse cells. The co-localization of H3K4me3 and H3K27me3 near the promoter region of genes is one of the most important epigenetic signatures of pluripotent cells ( Azuara *et al*., 2006 ; Bernstein *et al*., 2006 ; Sharov and Ko, 2007 ; Sachs *et al*., 2013 ). The importance of these domains relies on the fact that they localize to developmentally-regulated genes that are transcriptionally halted but can rapidly resolve upon differentiation by losing one of the marks and becoming expressed or silenced depending on the mark that they retain ( Tee and Reinberg, 2014 ). Interestingly, 44% of the bivalent genes detected in bovine ESCs were also present in human and mouse embryonic stem cells ( Mikkelsen *et al*., 2007 ; Pan *et al*., 2007 ). This percentage was equivalent to the number of genes from the mouse that are shared with the human species (52%) indicating that many of molecular features that delineate and specify the pluripotency state and early lineage commitment program are conserved across mammalian species ( Bogliotti *et al*., 2018 ). The similarities across species was also denoted in that the top gene ontology terms enriched in bivalent genes were shared between bovine ESC ( Bogliotti *et al*., 2018 ) and human ESC ( Li *et al*., 2013 ), including bivalent negative regulation of the canonical Wnt-signaling pathway, neuron migration, central nervous system development, and neuron differentiation.

Similarly, H3K4me3 was localized to a large set of genes (n=4,898) common to bovine, mouse and human ESCs, with a larger proportion of genes shared between human and bovine ESCs than between human and mouse ESCs ( Bogliotti *et al*., 2018 ).

Overall, these results indicate, that bovine ESC share the histone modification landscape of pluripotent cells from well characterized mammalian species; however, the paucity of information regarding the locus specific localization of histone modifications in bovine embryos prevents comparative analysis at this level.

## Concluding Remarks

In recent years, great advances have been made in our understanding of epigenetic remodeling mechanisms operating during preimplantation embryonic development. Discovery of conversion of 5-mC to 5-hmC by TET-enzymes and technological advances enabling detection and mapping of DNA methylation at single-base resolution throughout the genome starting from few to single cells has provided a much more complete picture of DNA methylation dynamics during preimplantation development, at least for mice and human embryos. The level of demethylation observed during preimplantation development is significant, but far from a complete erasure of the DNA methylation memory. Global methylation levels reach a minimum of about 30-40% CpG-methylation, or approximately half of that of the gametes and somatic cells (~ 80%). This methylation level is overshadowed by the demethylation level observed in PGCs of mice and humans, which achieve 3-6% methylation. Therefore, the greatest remodeling of epigenetic information seems to occur during germ cell formation, rather than after fertilization. The level of methylation reached after fertilization (half that of the gametes), together with the dynamics of demethylation, suggest that most of the methylation reduction could result from one round of DNA replication (maybe during the first cycle in PN-stage embryos) without maintenance of DNA methylation activity. At the male PN, replication dependent demethylation is facilitated by conversion of 5-mC to 5-hmC by TET proteins. Importantly, TET-dependent hydroxymethylation of 5-mC is also present in the female genome, although at a much lower level than the male one. And, some evidence for active removal of DNA methylation still exists, while at very low levels and the mechanism remains unclear. Demethylation of preimplantation development seems to be conserved between mouse and human embryos, while these two species differ in timing of EGA, and therefore suggest that DNA methylation remodeling may play a minor role in EGA or that human and mouse embryos may have different mechanisms in place that lead to EGA. Bovine embryos have an EGA timing similar to that of human embryos, but comparable information in terms of DNA methylation at base-specific level is not available. It will be interesting to determine the extent to which DNA methylation remodeling is conserved across other mammalian species.

Regarding histone modification remodeling, only recently some information about locus-specific dynamics of a few epigenetic marks has been produced in mouse embryos. This information produced unexpected interesting data that has not yet been fully understood, including the localization of H3K4me3 to broad domains, and lack of association of H3K27me3 marking and gene expression. Most other information about histone modifications is limited to overall levels determined by immunostaining studies and the overall role of some modifications and enzymes involved in their deposition/removal.

In view of the fact that genomic and epigenetic functional resources are getting better and more widely available, it is likely that a more complete and detailed picture of the molecular mechanisms of epigenetic remodeling during the preimplantation embryo development will arise. Such knowledge will likely result in our better ability to assess the impact of ART on embryos and progeny and to provide a basis for the “modification” of epigenetic information from animal embryos for improved production characteristics, as well as helping devise strategies for improved SCNT results.
